# Mechanism of neuroprotection by trehalose: controversy surrounding autophagy induction

**DOI:** 10.1038/s41419-018-0749-9

**Published:** 2018-06-15

**Authors:** He-Jin Lee, Ye-Seul Yoon, Seung-Jae Lee

**Affiliations:** 1 Department of Anatomy, Konkuk University, Seoul, 05029 Korea; 2 Research Institute of Medical Science, Konkuk University, Seoul, 05029 Korea; 3 IBST, Konkuk University, Seoul, 05029 Korea; 4 Departments of Medicine and Biomedical Sciences, Seoul National University College of Medicine, Seoul, Korea

## Abstract

Trehalose is a non-reducing disaccharide with two glucose molecules linked through an α, α-1,1-glucosidic bond. Trehalose has received attention for the past few decades for its role in neuroprotection especially in animal models of various neurodegenerative diseases, such as Parkinson and Huntington diseases. The mechanism underlying the neuroprotective effects of trehalose remains elusive. The prevailing hypothesis is that trehalose protects neurons by inducing autophagy, thereby clearing protein aggregates. Some of the animal studies showed activation of autophagy and reduced protein aggregates after trehalose administration in neurodegenerative disease models, seemingly supporting the autophagy induction hypothesis. However, results from cell studies have been less certain; although many studies claim that trehalose induces autophagy and reduces protein aggregates, the studies have their weaknesses, failing to provide sufficient evidence for the autophagy induction theory. Furthermore, a recent study with a thorough examination of autophagy flux showed that trehalose interfered with the flux from autophagosome to autolysosome, raising controversy on the direct effects of trehalose on autophagy. This review summarizes the fundamental properties of trehalose and the studies on its effects on neurodegenerative diseases. We also discuss the controversy related to the autophagy induction theory and seek to explain how trehalose works in neuroprotection.

## Facts

- Trehalose has been shown to be neuroprotective in animal models of various neurodegenerative diseases, such as Parkinson and Huntington diseases.
- Autophagy induction and aggregate clearance have been the primary hypothesis for the mechanism of neuroprotection by trehalose.
- Trehalose blocks autophagic flux from autophagosome to autolysosome in cell models.
- Trehalose may exert the neuroprotective effects through indirect mechanisms at the systemic levels, e.g., through influencing gut microbiota.

## Open questions

- What is the mechanism of neuroprotection by trehalose?
- How does trehalose block the autophagic flux?
- What are the effects of trehalose on gut microbiota?
- How does the chemical chaperone activity of trehalose influence on the neuroprotective functions?

## Introduction

**Fig. 1:**
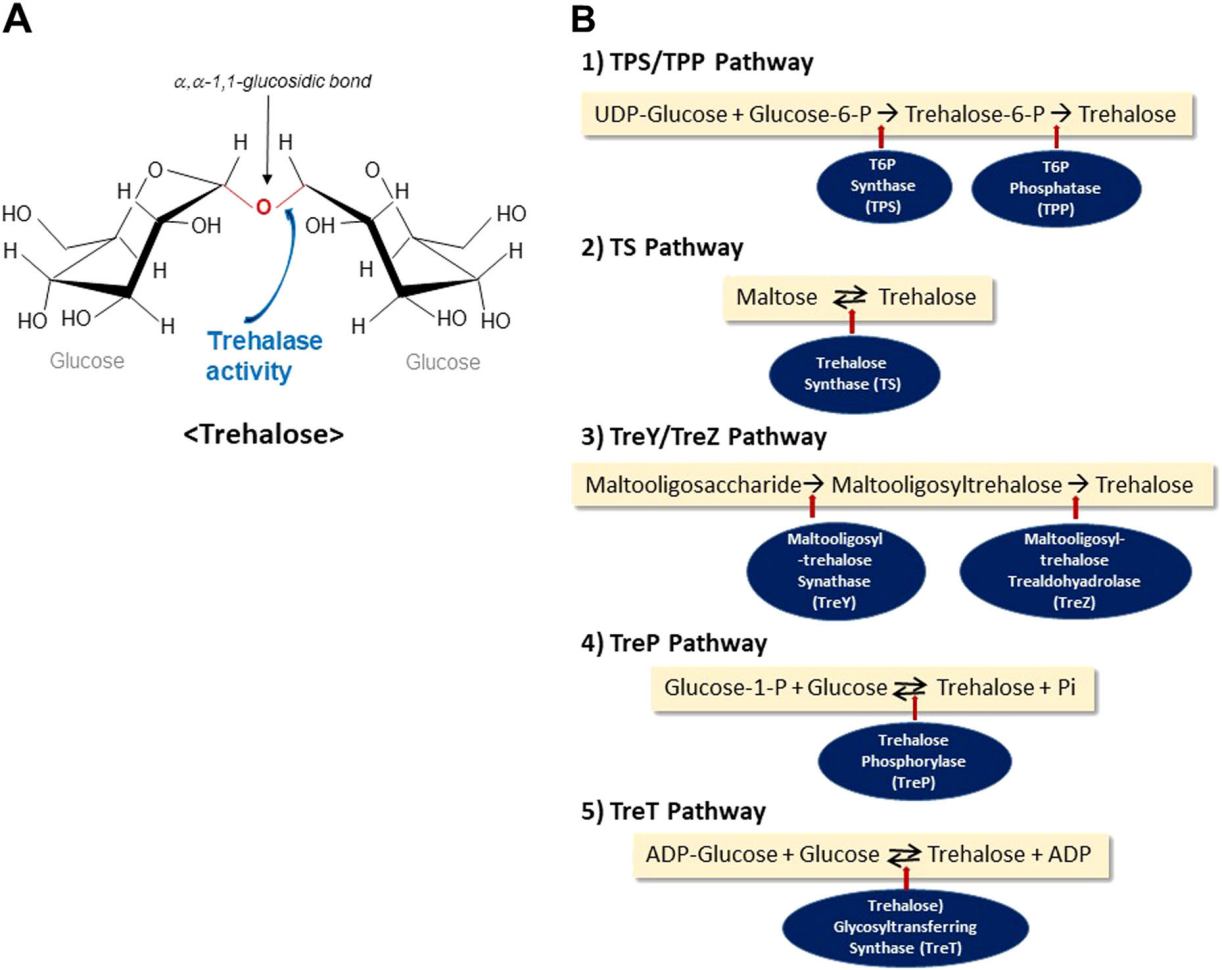
Trehalose metabolism. **a** Structure of trehalose. Trehalose consists of two glucose units linked through α, α-1,1-glucosidic bond. It is a stable non-reducing sugar, which is readily hydrolyzed by the enzyme trehalase. **b** Trehalose synthetic pathways. Five pathways to synthesize trehalose are shown. A most common pathway is the (1)‘‘TPS/TPP’’ pathway to form trehalose-6-phosphate, which is dephosphorylated to become trehalose. (2) Trehalose synthase (TS) synthesize trehalose from maltose. (3) Maltooligosaccharides are broken down to from trehalose by the TreY/TreZ pathway. (4) Trehalose phosphorylase (TreP) utilizes glucose-1-phosphate to form trehalose. (5) ADP-glucose is used to from trehalose by trehalose glycosyltransferring synthase (TreT).

Trehalose (O-α,-d-glucopyranosyl-[1 → 1]-α-d-glucopyranoside) is a disaccharide comprised of an α, α-1,1-glucosidic bond between two α-glucose units (Fig. 1a). It is a non-reducing stable sugar, which is not readily hydrolyzed by acid or α-glucosidase. Its inert characteristic ensures that it does not readily interact with proteins or other biomolecules^1,2^.

Trehalose is detected in most organisms except for vertebrates^3^. Not one gene involved in trehalose biosynthesis nor storage is found in vertebrate genomes^3^. Why do vertebrates not synthesize trehalose? Rather than losing the ability to produce trehalose in the evolution process, it seems that they never acquired such capacity in the first place. Vertebrates and invertebrates have strikingly divergent ancestors and follow separate lines in the early steps of evolution. Most invertebrates come from protostomes, whereas vertebrates and some invertebrates, such as Echinodermata, are originated from deuterostomes. Deuterium-derived primitive organisms also do not own trehalose-synthesizing genes^3^.

Prominent features of trehalose arise from its non-reducing property, which leads to high hydrophilicity, chemical stability, and strong resistance to acid hydrolysis and cleavage by glucosidases. Furthermore, trehalose was shown to act as a molecular chaperone to help refold partially denatured proteins^4,5^.

Recent reports of trehalose as an autophagy inducer and a protector against pathological changes in various models of neurodegenerative disorders suggested this disaccharide as an attractable therapeutic option. This review provides a careful assessment of these studies and discusses the potential mechanism of neuroprotection by trehalose.

## Structure and biochemical characteristics of trehalose

The chemical stability of trehalose arises from the 1,1-glycosidic linkage, which has low energy (1 kcal/mol) compared to other similar disaccharide sucrose (27 kcal/mol). It is not readily hydrolyzed into glucose units unless the enzyme trehalase is present^6^. The glycosidic bond in trehalose has greater flexibility than in other disaccharides, and it facilitates the sugar to conform with other polar groups of biomolecules easily^2^. Trehalose has the highest ability for hydration compared to other sugars. As a result, it may enhance stabilization of membrane lipids by arranging the water molecules nearby or by direct interaction with polar biomolecules in replacement of water molecules^7,8^.

There are three suggested mechanisms by which trehalose stabilizes proteins: water replacement, glass transition, and chemical stability^2^. Trehalose inhibits protein denaturation by the exclusion of water molecules from the surface of proteins when cells are in the dehydrated condition^9^. In the dry state, it maintains proteins in the folded state by replacing water molecules and forming hydrogen bonds directly with proteins^10^. The unique property of trehalose to create a stable non-hygroscopic glass at high temperatures in the dry state also allows maintenance of the protein structure^11^. The amount of trehalose accumulation in different yeast species is related to the ability to survive heat and dehydration^12,13^. In nematodes, trehalose accumulates at the onset of dehydration^14^. Trehalose is rapidly broken down once the stress is relieved, bringing it down to the normal level.

Trehalose, therefore, acts as a natural stabilizer of life processes, withstanding extreme temperatures, nutrient deprivation, osmotic pressures, and dehydration in many species of invertebrates^1,12,13,15^. Trehalose serves as an excellent desiccant for many organisms. Even human primary fibroblasts, artificially producing trehalose, could be maintained in the dry state for up to 5 days^16^.

## Trehalose in invertebrates

### Energy source

Trehalose is synthesized in most organisms of prokaryotes and eukaryotes except for vertebrates. It provides extra energy needed during certain stages of development in anhydrobiotic organisms^15^. Some bacteria spores accumulate trehalose up to ~25% of the dry weight of the spore^12^. Bacteria can use trehalose as an exogenous carbon source as well as a structural component of the cell wall^3^. In yeast and fungi, it is reserved during dormancy^17^. In growing plants, trehalose is the major disaccharide present^18^. It is the primary sugar in the hemolymph, constituting 80–90% of total sugars^19^. It is also an important energy source for flight in insects^15^.

### Trehalose metabolism

There are five known biosynthetic pathways (Fig. 1b) for trehalose^20^. First, the most common pathway, ‘‘TPS/TPP’’ pathway, used by trehalose-synthesizing organisms utilizes trehalose-6-phosphate (T6P) synthase (TPS)^21^. The glucose unit from UDP-glucose is transferred to glucose-6-phosphate by TPS and forms T6P. It is then dephosphorylated by trehalose-6-phosphate phosphatase (TPP) to generate trehalose. Second, trehalose synthase (TS), in some bacteria such as *Pimelobacter sp*, *Pseudomonas syringae*, and *Thermus caldophilus*, intramolecularly arranges maltose, another disaccharide with two glucose units (α-(1,4)), into trehalose (α-(1,1))^22^. Third, the ‘‘TreY-TreZ’’ pathway in some bacteria converts maltooligosaccharide-containing starch or glycogen into trehalose^23^. Fourth, trehalose phosphorylase (TreP) either hydrolyzes trehalose into glucose-1-phosphate and glucose or acts reversibly depending on the species^24^. And finally, Trehalose glycosyltransferring synthase (TreT) forms trehalose from ADP-glucose and glucose in some primitive bacteria^25^.

### Development

In plants, trehalose regulates growth and development^18^. Mutants impaired in producing trehalose-6-phosphate (T6P) could not complete embryogenesis in *Arabidopsis thaliana* (*A. thaliana*)^26^. Levels of T6P also controls flowering phase in the plant^27,28^.

Trehalose is the primary sugar crucial for insect growth and development, representing about 20% of the total carbohydrate pool in certain stages of development^3^. *C. elegans* could store trehalose up to 2.5% dry weight in a specific stage of the life cycle, highest concentrations found in eggs and dauer larvae stages^3^.

### Trehalose glycolipids

Trehalose exists as a α,α-trehalose diester forms in bacteria, such as Mycobacteria and Corynebacteria, and in *C. elegans* dauer larvae. These trehalose glycolipids are considered to have roles in protection against harsh environmental conditions. They are first discovered in *Mycobacterium tuberculosis* and referred to as ‘‘cord factors,’’ the term initially coined to express their crucial functions in the pathogenesis of the bacteria^29^. These ‘‘cord factors’’ are later turned out to be trehalose dimycolates^30,31^. The second class of trehalose glycolipids is sulfolipids in the forms of sulfate esters^32^. Trehalose glycolipids are incorporated in the membrane wall of bacteria and exposed to extracellular space. They bind and activate macrophages through the FcRγ-Syk-Card9-dependent pathway. The biological functions of trehalose glycolipids include infectivity, anti-tumor effects, adjuvant function, and antibacterial activity, and the ability to induce granuloma and angiogenesis^33^.

### The symbiosis of plant–microorganism, plant–insects

Most bacteria and fungi produce trehalose, and some of them rely on trehalose metabolism for infectivity. *Magnaprothe oryzae*, a fungus causing rice blast disease, loses its pathogenicity when *TPS1* gene is deleted^34^. *Pseudomonas aeruginosa* strain PA14 is a multi-host pathogen that infects nematodes, insects, plants, and vertebrates. A mutant strain that lacks trehalose-synthesizing activity is unable to infect *A. thaliana*, but not other non-plant hosts^35^.

The increase of extracellular trehalose in the plant may be a sign of infections of insects, nematodes, or parasitic plants^36–39^. Plants need to prepare for such attack by activating defense mechanism. These interactions between plants and other organisms may have considerable effects on the plant’s own trehalose metabolism^18^.

### Stress responses

Trehalose is present in mycobacteria and corynebacteria cell walls, where it is considered to play a structural role. In many bacteria, trehalose is involved in adaptive responses to osmotic stress and extreme temperatures^20,40,41^. Yeast utilizes trehalose as a carbon source and to respond to abiotic stresses^42–44^.

Higher organisms, such as plants and insects, utilize trehalose in response to anhydrosis, osmotic stress, and extreme temperatures^45,46^. In plants, accumulation of trehalose under such stress is related to transcriptional activation of genes in trehalose biosynthesis or by inactivation of trehalase enzyme^47^.

### Chemical chaperone

Trehalose stabilizes native proteins and preserves membrane integrity during stresses in vitro^2,6^. Yeast accumulates trehalose during heat shock to protect against thermal denaturation and aggregation of proteins, but at high concentrations, trehalose inhibits reactivation of denatured proteins by molecular chaperones^4^.

### Trehalase, the hydrolyzing enzyme

Three yeast trehalases, neutral trehalase 1 (NTH1), NTH2, and acid trehalase (ATH1), have been identified to date. NTH1, a cytosolic protein with functional enzymatic activity, is primarily responsible for hydrolysis of trehalose. NTH2 is a homolog of NTH1 but lacks enzymatic activity. ATH1 is a vacuolar protein, which is active at pH 4.5. It is activated during cellular stresses. Mutation in the *NTH1* gene accumulates trehalose inside the cells at high levels, but the cells’ ability to survive and recover from extreme heat is impaired, suggesting that trehalose levels during stress must be tightly controlled for life processes^48,49^.

## Trehalose in vertebrates

### Trehalose metabolism

Vertebrates do not synthesize or store trehalose, but retain active hydrolyzing enzyme, trehalase, in the small intestine^15^. Trehalase resides in specific locations, such as intestinal mucosa and renal brush-border membranes, liver, and possibly blood^50^. Vertebrates express the enzyme during gestation stage. The highest concentration is reached after weaning. The levels of trehalase from birth remains throughout adult life^51^. Intestinal trehalase is responsible for rapid degradation of ingested trehalose. Various organisms that constitute the human diet, including plants and fungi, contain trehalose. Like in lactose intolerance, having a low concentration of trehalase causes malabsorption, diarrhea, or other gastrointestinal symptoms^52^. Intake of probiotic *Saccharomyces boulardii* by such patients had increased trehalase activity in the intestine and reduced those symptoms^53^.

Urinary trehalase has been proposed to be a specific marker for kidney damages. In diabetes, higher trehalase activity and genetic variations in the trehalase gene were noted^54,55^. Human trehalase (TREH) has a remarkable feature shared with yeast acid trehalase 1 (ATH1)^56^. TREH rescued phenotypes of yeast ATH1 mutant but not NTH1 or NTH2. ATH1 is present in the vacuole and catalyze the hydrolysis of extracellular trehalose^57,58^. These results suggest that human trehalase gene *TREH* may act as a stress-response gene involved in the utilization of exogenous trehalose^56^.

### Trehalose in neuroprotection and protein aggregation

Saccharides in glycoproteins and glycolipids play essential roles in the brain. They assist in brain development, synaptogenesis, synaptic transmission, and neurotransmitter production^59,60^. Moreover, several saccharides enhanced brain functions in animal and human studies. Dietary polysaccharides derived from yeast and plants improved cognitive function and mood in healthy young and middle-aged human adults^61–63^.

Neuroprotective properties of trehalose were mentioned in animal studies. Growing *C. elegans* in trehalose-containing growth medium extended the lifespan^64^. Mouse models with neurologic defects partially recovered from their behavioral and neurobiological defects^65^. Oral administration of trehalose improved motor dysfunction and extended the lifespan of a mouse transgenic (tg) model of Huntington disease (HD)^66^. Superoxide dismutase 1 (SOD1) mutant tg mice had a significantly prolonged lifespan and enhanced neuronal survival with trehalose administration^67^. Parkin^-/-^/Tau^VLW^ mice had shown significant reductions in the phosphorylated tau-positive neuritic plaques and astrogliosis in the brain^68^. In 1-methyl-4-phenyl-1,2,3,6-tetrahydropyridine (MPTP) mouse model of Parkinson’s disease (PD), trehalose inhibited the reduction in the striatal dopamine levels and prevented gliosis^69^.

### Evidence for trehalose as an autophagy activator and an inhibitor of protein aggregation

The initial observations of trehalose as a neuroprotective reagent in human and animal studies led to the following in vitro and in vivo studies. In a yeast study, it was first described as a potential inhibitor for the aggregation of denatured proteins^4^. Not only did trehalose directly stabilize proteins in the native state but it also reduced aggregation of proteins that have already been denatured. In HD tg mouse model, trehalose decreased polyglutamine aggregates in cerebrum and liver^66^. In vitro aggregation of Aβ peptides was also inhibited in the presence of trehalose^70^. When trehalose was orally administered to SOD1 mutant tg mice, there was a decrease in the accumulation of SOD1 aggregates in the brain^67^.

**Fig. 2:**
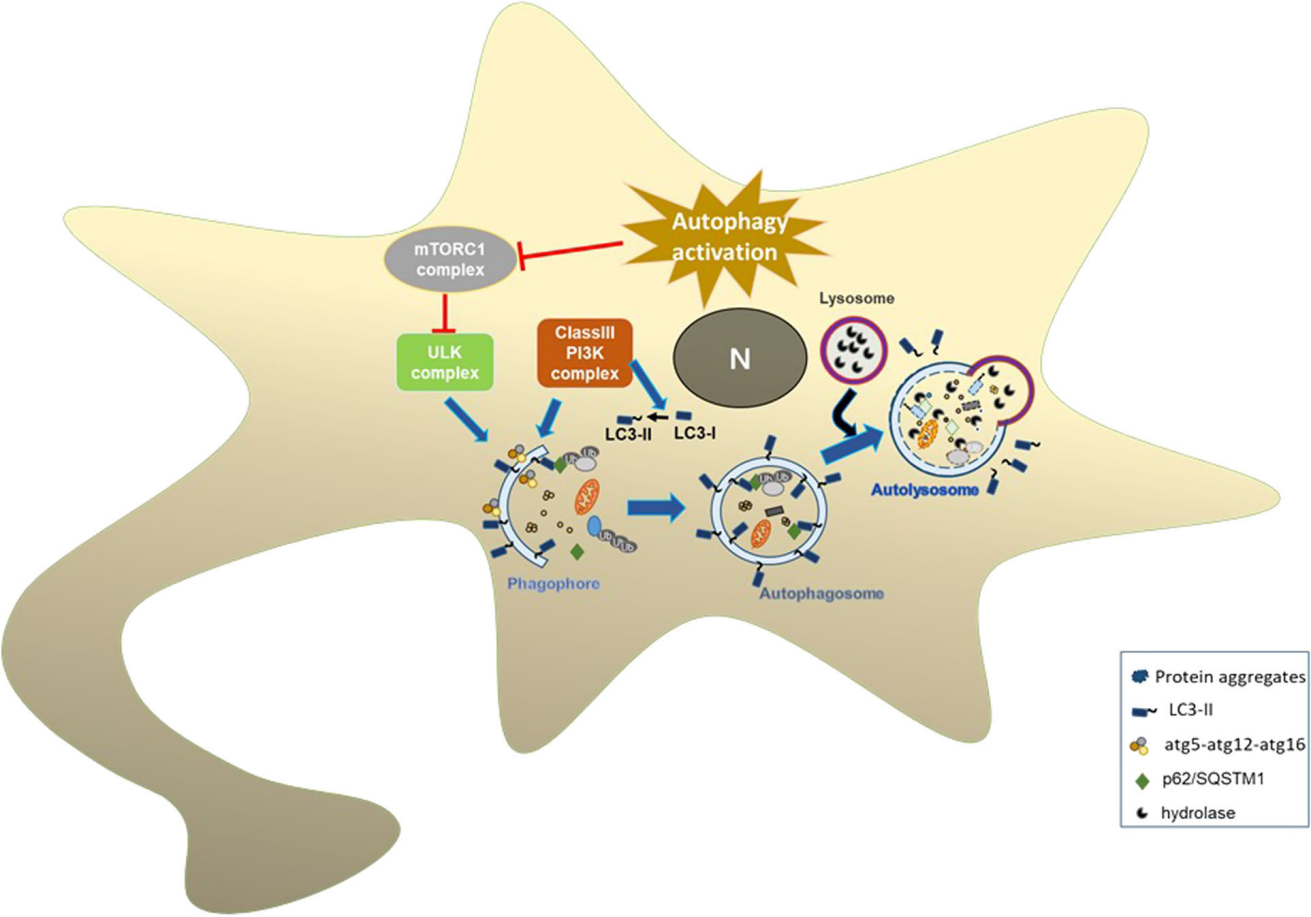
Autophagic pathways. Activation of autophagy inhibits mTORC1 complex that leads to autophagosome formation. Atg5–atg12–atg16 complex helps elongation of phagophore membrane. Cytosolic LC3-I is converted to lipidated LC3-II and binds to inner and outer membranes of autophagosomes. Mature autophagosomes are formed and are ready for fusion with lysosomes. Lysosomal hydrolases degrade autophagosome contents.

Meanwhile, several reports proposed that trehalose might induce autophagy^71^. Autophagy is a lysosome-mediated degradation process to remove damaged cellular components. These include damaged organelles, such as mitochondria, endoplasmic reticulum (ER), and peroxisomes, as well as misfolded or aggregated proteins and intracellular pathogens^72^. Three major types of autophagy have been described so far, which are macroautophagy, chaperone-mediated autophagy (CMA), and microautophagy^73^. Impairment of autophagy is linked to many diseases including cancer, inflammatory diseases, and neurodegenerative disorders. Environmental stresses, such as starvation, growth factor depletion, and oxidative stress, activate macroautophagy through inhibition of mTOR. Such stresses lead to transcriptional activation of autophagy genes and downstream activation of autophagosome synthesis (Fig. 2).

In a study where trehalose was treated to the prion-infected cells, LC3-II levels increased, suggesting that autophagy was activated^74^. De novo production of PrP^Sc^ aggregates decreased, and their subcellular localizations changed in the same study. Oral intake of trehalose in Parkin^-/-^/Tau^VLW^ mice resulted in a significant reduction of p62/SQSTM1, an autophagic substrate, in the striatum, where dopamine levels were reduced. Trehalose prevented cell death and reduced polyubiquitinated proteins that were induced by epoxomicin, a proteasome inhibitor^75^. Epoxomicin-induced autophagic inhibition was also relieved by trehalose, and the LC3-II and p62/SQSTM1 returned to normal levels. In α-synuclein tg mice, oral intake of trehalose, not an intraperitoneal injection, induced autophagy and reduced α-synuclein aggregates^76^.

These studies supported the hypothesis that trehalose acts as an autophagy inducer, and the neuroprotective effects of trehalose may be a consequence of autophagy activation, which leads to the clearance of toxic protein aggregates.

### Is trehalose a direct autophagy inducer?

While quite a few studies proposed that trehalose is an autophagy inducer, there are some limitations in these studies, questioning the validity of the hypothesis. Most of these studies have not attempted to distinguish between autophagy induction and inhibition of autophagic flux, both of which could increase LC3-II levels. When autophagy is initiated, the autophagosomes form in the cytosol. Cytosolic LC3 (LC3-I) is converted to lipidated LC3 (LC3-II), which binds to both inner and outer membranes of autophagosomes (Fig. 2). Mature autophagosomes then fuse with lysosomes and the autophagosome contents, including LC3-II in the inner membrane, are degraded by lysosomal enzymes. Therefore, autophagy flux would begin with a transient elevation of LC3-II levels, but then turn over of LC3-II as the autophagy flux continues will eventually lead to the steady-state levels of LC3-II proteins in the cells^77^. Successful completion of autophagy would also decrease levels of p62/SQSTM1, a receptor for polyubiquitinated autophagic substrates.

In a study by Sarkar et al.^71^, trehalose was reported to have an autophagy-inducing effect, showing increased levels of LC3-II. Several other studies also observed an elevation of LC3-II levels by western blotting or immunocytochemistry^75,78^. Since an increase in autophagosome formation and blockage of autophagic flux could both lead to a rise in LC3-II levels, an increase in the number of GFP-LC3 puncta is not sufficient to presume that autophagy induction has occurred.

**Fig. 3:**
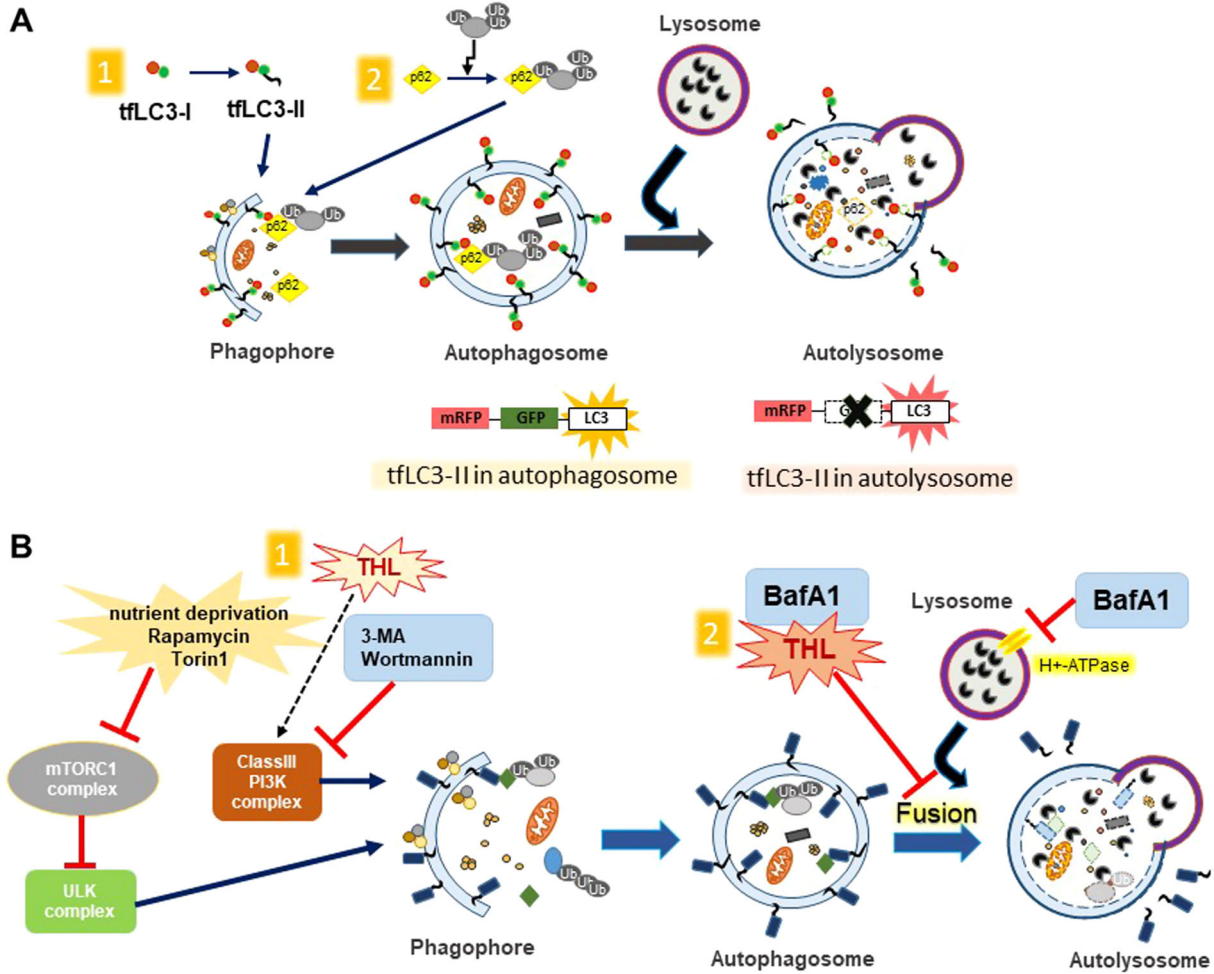
Regulation of autophagy. **a** Distinguishing between autophagosomes and autolysosomes. mRFP-GFP tandem fluorescent-tagged LC3 (tfLC3) fluoresces both GFP and RFP signals (yellow) before it is delivered to lysosomes. GFP in tfLC3 loses its fluorescence in the acidic and degradative lysosomal environment (red). Autophagy induction increases autophagosomes (yellow) and autolysosomes (red) together because the autophagic flux to lysosomes is not disturbed. Blocking fusion of autophagosomes and lysosomes, however, would increase the number of autophagosomes (yellow) only. **b** Autophagy modulating factors. Autophagy is initiated through inhibition of mTORC1 complex and activation of Class III PI3K complex. 3-methyladenine (3-MA) and Wortmannin prevent autophagy through inhibition of mTORC1 complex. Trehalose may activate autophagy through PI3K. BafA1 and trehalose could both inhibit fusions of autophagosomes and lysosomes, thus blocking final stage of autophagy

To circumvent such issue, mRFP-GFP tandem fluorescent-tagged LC3 (tfLC3) is now widely in use^79^. tfLC3 fluoresces both GFP and RFP signals before the fusion with lysosomes. However, GFP loses its fluorescence once delivered to acidic and degradative lysosomal environment. When the tfLC3 proteins in autophagosomes reach lysosomes, the GFP fluorescence weakens, and only RFP signals remain. Therefore, the increase of autophagosomes by autophagy induction would show elevations in both autophagosomes (yellow fluorescence) and autolysosomes (red fluorescence) because the autophagic flux to lysosomes is not disturbed (Fig. 3a). However, an increase of autophagosomes by blockage of autolysosome formation would show an elevated number of autophagosomes (yellow) but no autolysosomes (red). In our latest study, we have utilized tfLC3 to distinguish detections of autophagosomes and autolysosomes and analyzed effects of trehalose on autophagy flux. Our results showed that, with trehalose treatment in SH-SY5Y neuronal cells, the number of autophagosomes but not autolysosomes increased, suggesting that autophagic flux is inhibited^80^. Another recent study also showed using tfLC3 that trehalose treatment caused inefficient delivery of LC3 from autophagosomes to lysosomes in H4 glioma cells^81^. Examining levels of autophagy substrates, such as p62/SQSTM1, is another way of assessing successful accomplishment of autophagy process. Completion of autophagy would lead to the degradation of the autophagy substrates. In studies of Parkin^-/-^/Tau^VLW^ and SOD1 tg mice, animals fed with trehalose-containing water showed decreased levels of p62/SQSTM1 protein in the striatum and spinal cord motoneurons, respectively^67,68^. However, in contrary to the observations in animal studies, trehalose led to substantial accumulations of both LC3-II and p62/SQSTM1 in H4, HegG2, and 293 cells^81^. These studies in cell models raised a question as to whether trehalose is a direct autophagy inducer, and opened up the possibility that it may act as a blocker of autophagic flux. Our recent study also showed the similar results that both LC3-II and p62/SQSTM1 levels increased with trehalose treatment in cells^80^.

Bafilomycin A1 (BafA1), a vacuolar-type H^+^ ATPase inhibitor, is often used to inhibit the late stage of autophagy process where autophagosomes fuse with lysosomes. LC3-II levels increase with BafA1 treatment. Chloroquine, a lysosomotropic agent, also prevents autophagosome–lysosome fusion and inhibits autophagy. A study investigated the effect of BafA1 on trehalose-modified autophagy^71^. COS-7 cells, transfected with Htt proteins, were treated with trehalose (100 mM, 48 h) and BafA1 (200 nM, 4 h). The results showed that LC3-II levels increased more when BafA1 and trehalose were treated together than each reagent treated alone. In a different study, ScN2a neuronal cells treated with trehalose (100 mM, 48 h) and BafA1 (200 nM, 4 h) showed similar results that the LC3-II levels were increased more with treating both reagents together than with treating each alone^78^. These results were main evidence used as evidence for trehalose being an autophagy activator.

However, the use of BafA1 as an autophagy blocker raised a concern when treated for a short time ( < 6 h), which might be insufficient to block autophagy entirely^82^. Temporal examination of BafA1 showed that short-term treatments with BafA1 resulted in impaired acidification of lysosomes, but only after treatments with extended periods prevented the fusion between lysosomes and autophagosomes. Therefore, it was recommended that BafA1 should be treated for an extended period ( > 6 h for most cell types) to inhibit autophagic flux. In our recent work, we have also performed similar experiments but treated BafA1 for a longer time (12 h or more)^80^. The results showed that the effect of trehalose and BafA1 together was similar to BafA1alone, suggesting the role of trehalose as an inhibitor of autophagic flux.

Recent studies on trehalose aimed to uncover molecular pathways of autophagy activation and neuroprotection were reported. Trehalose prevented glucose/fructose uptake by inhibition of solute carrier 2A (SLC2A) proteins and reduced hepatic steatosis^83^. Impairment of glucose uptake has the same effect as the starvation, which affects autophagy.

Little is known as to how cells take up trehalose. SLC2A8 (GLUT8) was presented as a mammalian trehalose transporter, partly responsible for uptake of trehalose and trehalose-induced autophagy in hepatocytes^84^. However, trehalose-induced LC3-II accumulation in N2A neuroblastoma cells was not SLC2A8-dependent, because SLC2A8 is not localized in the plasma membrane in neuronal cells. These studies suggest that trehalose uptake mechanism may be different in neuronal cells compared to hepatocytes and may act differently on the autophagic pathway.

In summary, unlike the observations in animal models where trehalose administration seems to induce autophagy in the brain, trehalose seemed to be more of an autophagic flux blocker than of an autophagy inducer when treated directly to cultured cells, including neuronal cells^80^. tfLC3 staining and immunoblotting of autophagic markers, LC3-II and p62/SQSTM1, support the conclusion that trehalose may impair lysosomal activity and interfere with degradation through autolysosomes, acting similarly to lysosomotropic inhibitors (Fig. 3b).

### Trehalose effects on other pathways

Autophagy has received the most attention as the key player in the mechanism of action of trehalose in mammalian cells. However, there have been several studies suggesting its roles in other cellular processes. Trehalose downregulated PARP-1 and PARP-2 expression after lipopolysaccharide (LPS) and interferon gamma (INFγ)-induced oxidative stress in primary rat astrocyte and oligodendrocyte cultures, suggesting the anti-apoptotic function of trehalose under oxidative stress conditions^85^. Trehalose intake by Lewy body disease model mice increased levels of several chaperone molecules, such as HSP90 and SigmaR1, suggesting its roles in protein folding^76^. Oxygen-glucose deprivation (OGD) inhibits proteasome activity via suppression of both oxidative stress and ER stress. Trehalose inhibited OGD-induced autophagy while preserving proteasome activity^86^.

Trehalose may also regulate stress granules, which are RNA–protein complexes in the cytoplasm of eukaryotic cells, produced under specific stress conditions. The function of these stress granules is thought to protect RNAs from degradation, thereby preventing cell death and stress signaling. The stress granule formation is initiated by recruitment of eukaryotic initiation factor 2 (eIF2) to form eIF2-GTP-tRNAiMet ternary complex. Many proteins in the stress granules are dysregulated in human diseases, such as ALS. Prolonged accumulation of the stress granules may lead to increased protein aggregation and the pathogenesis of neurodegenerative diseases. Trehalose efficiently promoted the stress granule disassembly via the p-eIF2α pathway, suggesting that neuroprotective effects of trehalose may include the regulation of the stress granules^87^.

### Does trehalose directly affect clearance of protein aggregates?

Neuroprotection and autophagic induction by trehalose led to a hypothesis that trehalose promotes aggregate clearance through activation of autophagy. Although several studies attempted to link the two processes together, the evidence provided has not been conclusive. In this section, we will critically assess the literature on this subject and examine whether trehalose can directly promote aggregate clearance.

Sarkar et al.^71^ reported first that trehalose has an autophagy-inducing effect and enhanced clearance of protein aggregates, such as mutant huntingtin and α−synuclein in cultured cells. A similar observations were made where trehalose treatment reduced polyubiquitinated protein aggregates that were induced by epoxomicin^75^. The same study also showed the reduced tau and α−synuclein aggregates in NB69 neuroblastoma cells. In these studies, however, the quantitative analysis of the aggregates might have been limited. For example, α−synuclein immunoblotting only displayed the reduction of monomers. The high molecular weight α−synuclein aggregates were not shown. In the latter study, the amounts of phosphorylated tau were not normalized to the total tau levels (tau-5 antibody), and both phosphorylated and total tau seems to change in similar patterns. In the case of polyQ proteins of the former study, however, immunoblotting showed the high molecular weight protein aggregates left in the wells of sodium dodecyl sulfate polyacrylamide gel electrophoresis gels. Further characterization of the aggregates with different methods would have strengthened the argument on the role of trehalose in aggregate clearance.

Another study by Tanaka et al.^66^ examined the effect of trehalose on polyglutamine aggregates in the Neuro2A mouse neuroblastoma cells expressing a GFP fused polyQ protein. Trehalose and other small molecules were delivered into cells directly by a lipid-based transient cell permeabilization method. The result showed a reduction in the number of cells with aggregates and improvement in cell viability upon trehalose treatment. However, using an artificial cell delivery system might have allowed high levels of trehalose in cells that might not occur in vivo.

In a different study, cells stably infected with prion and treated with trehalose displayed more diffuse and dispersed patterns of PrP^Sc^ staining in the periphery of the cells compared to control cells which had compact, round inclusion stainings in the perinuclear area^74^. One thing that was noticeable in this study is that trehalose was treated at low concentrations (~50 μM), compared to 50–100 mM typically used in other studies. Further characterizations of the compact/round and dispersed structures would help determine whether these structures represent protein aggregates or merely represent limited localization of PrP^Sc^ to specific organelles of the cell. Trehalose treatment for longer incubation period (up to six passages) did not modify proteinase K (PK) resistance of PrP^Sc^ aggregates in this study, suggesting that trehalose may not affect protein aggregation at low concentrations.

Interestingly, several recent works reported the results that contradicted the role of trehalose in aggregate clearance. Partially denatured proteins after heat shock were efficiently refolded in the presence of trehalose, thereby suppressing aggregation in yeast^4^. However, the continued presence of trehalose interfered with protein refolding in yeast. A study with amyloid precursor protein (APP) showed that trehalose decreased degradation of APP and other long-lived proteins in cells^81^. These effects are associated with diminished lysosomal hydrolase activities, such as cathepsin D. In our latest study, addition of trehalose into the culture medium of SH-SY5Y cells increased α-synuclein aggregation and lysosomal integrity was also impaired^80^. Curiously, there was little correlation between protein aggregation and cell toxicity when trehalose was present, suggesting that the aggregates formed under trehalose treatment conditions were not toxic or that trehalose protected cells from aggregate toxicity. Mouse primary cortical neurons exposed to pre-formed fibrils (PFF) of α-synuclein had shown an increased abundance of phosphorylated S129 form and reduced cell viability^88^. Trehalose failed to remove α-synuclein aggregates in these cells. However, it increased basal cell viability compared to non-treated cells. These results suggest that protective effects of trehalose may act independently from its effects on protein aggregation.

## Direct vs. indirect mechanism of neuroprotection by trehalose

Studies have shown that administration of trehalose is neuroprotective in animal models. When trehalose was administered, animals with neurodegenerative diseases lived longer with reduced neuropathology and alleviated behavioral phenotypes. These animal studies also exhibited autophagy activation and the reduction of protein aggregates. Inflammatory responses decreased, and gliosis diminished in response to trehalose. More neurons survived in the specific areas of the brain in the disease models. In cells, however, the connection of autophagy activation and the reduction of protein aggregates to trehalose is still controversial. Careful analysis of autophagy flux and protein aggregates suggested that unlike the previous hypothesis, trehalose interferes with autophagy flux and increases, rather than decreases, the levels of protein aggregates in cultured cells. These results raised the possibility that the cause of autophagy induction and aggregate clearance in animal models of neurodegenerative diseases may not stem from the direct effects of trehalose on neurons. The neuroprotective effects of trehalose in animals may be indirect.

What might be the mechanism of neuroprotection by trehalose? Contrary to the direct uptake of trehalose in culture, trehalose treated to animals in drinking water is likely to be hydrolyzed by trehalase enzyme in the gut. Even if some trehalose enters the blood stream, there is the blood–brain barrier (BBB) that limits the access of trehalose to the brain.

One possibility is that the effects of trehalose are exerted at the gut level. For example, trehalose may influence gut microbiota. Trehalose can protect microbes from harmful stresses and enhance the survival. Increasing body of evidence has accumulated over the years that gut microbiota has wide-spread effects on many physiological systems, including the central nervous system, raising the possibility that trehalose exerts its neuroprotective roles through microbiota-gut-brain signaling^89^. Consistent with this hypothesis, only the oral intake of trehalose, not an intraperitoneal injection, efficiently induced autophagy in the mouse brain, suggesting that the neuroprotective effects of trehalose require the gastrointestinal (GI) system^76^.

**Fig. 4:**
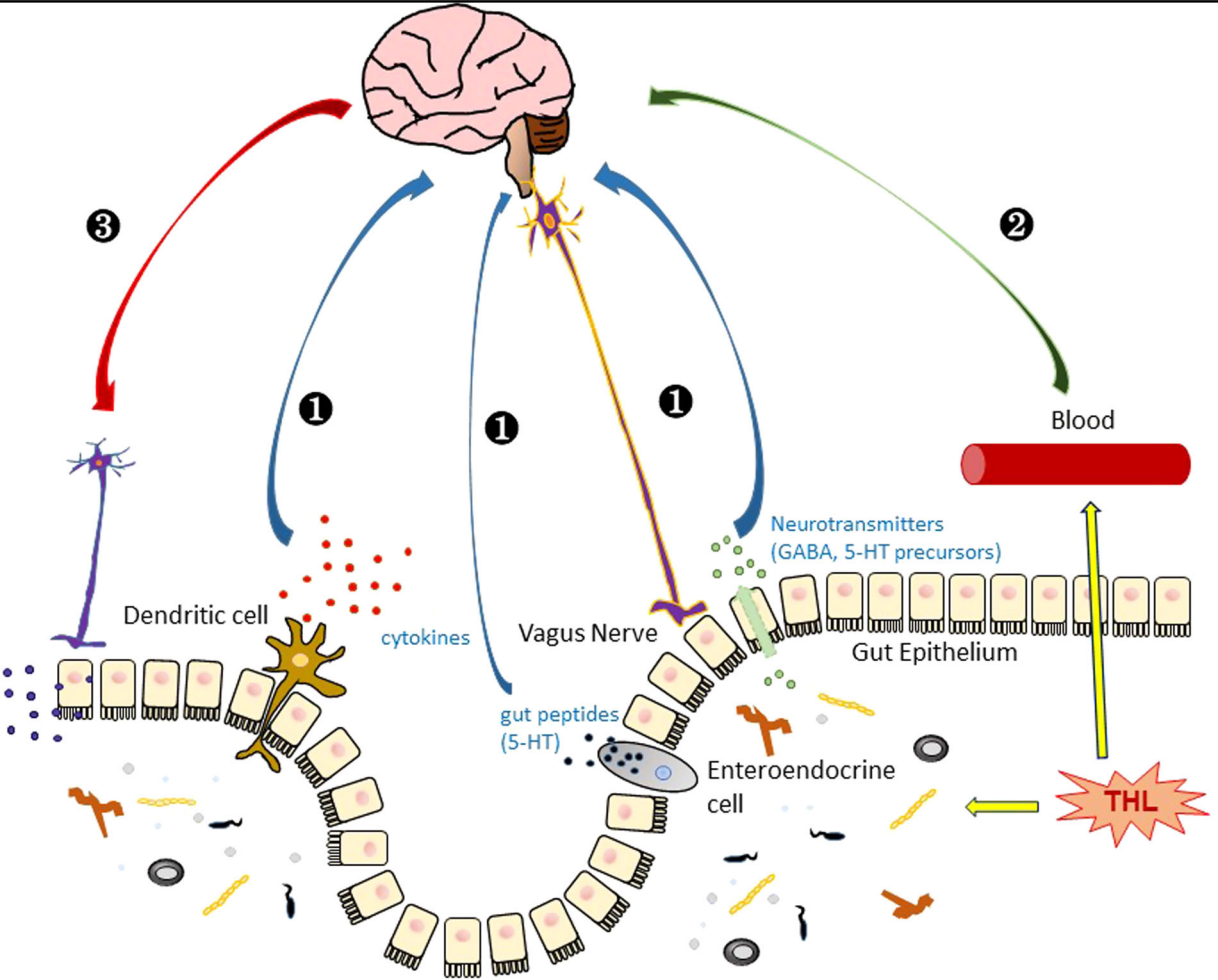
A schematic view of a hypothesis of trehalose function in the brain. (1) Trehalose indirectly affects brain function through the regulation of gut microbes, which sends signals to the brain by dendritic immune activation or secretion of neurotransmitters and gut peptides that may be delivered through vagus nerve to the brain^91^. (2) Direct transport of trehalose to the brain, which passes through the blood–brain barrier and affects neuronal cells. (3) The brain sends signals to the enteric system to modulate trehalose function

On the other hand, one cannot rule out the possibility that trehalose travels through the blood stream and enter the brain, exerting its neuroprotective functions directly to neurons. There are reports of trehalose detection in blood plasma and its relation to diabetes^54,55^. It is also detected in liver and kidney, but the functions of trehalose in these organs are still unknown. A recent study by Martano et al.^90^ detected trehalose in mouse brain, particularly in the hippocampus and cortex. Endogenous trehalose was detected in both astrocytes and neurons, but the hydrolyzing enzyme, trehalase, was localized in only neurons. Astrocytes were able to take up and release trehalose into the extracellular space. The source of trehalose, however, was unclear as trehalose-synthesizing enzymes are not present in vertebrates. These results raise a possibility that trehalose may travel from gut to brain, through blood or other carriers, and act directly on neurons. Once trehalose reaches the brain, it may act directly on neurons and other cells to affect protein folding, act as a signaling molecule to activate stress responses, or regulate autophagy and cell death mechanisms. However, it is doubtful that the concentrations of trehalose in neurons and other brain cells reach the levels at which most of the in vitro studies have been done (Fig. 4).

## Conclusion

Neuroprotective effects of trehalose have been fairly consistent in many different neurodegenerative disease models. It has been hypothesized that trehalose directly acts on neurons and induce autophagy, thereby promoting the clearance of protein aggregates. However, careful examination of the literature raised a question as to whether trehalose can directly induce the autophagic process. A recent paper, indeed, showed that trehalose treatment of neuronal cells hampers the progression of autophagosomes to autolysosomes. Furthermore, it is not clear whether and how much trehalose can be delivered to the brain parenchyma when it is administered to the animals. We propose that trehalose intake exerts neuroprotective effects in neurodegenerative disease models through a direct or indirect mechanism, which may involve the gut microbiota. Future studies aiming to resolve the mechanism of trehalose-mediated neuroprotection may pave the ways to the development of novel therapy for neurodegenerative diseases.
